# Pathological Mechanism and Clinical Therapy Progress of Schlemm's Canal

**DOI:** 10.1155/2024/9978312

**Published:** 2024-10-26

**Authors:** Yasha Zhou, Zhenxin Liu, Wenyong Gao, Yijing Yang, Qinghua Peng, Hanyu Tan

**Affiliations:** ^1^Hunan University of Chinese Medicine, Changsha 410208, Hunan, China; ^2^Ubon Ratchathani University, Warin Chamrap, Ubon Ratchathani 34190, Thailand; ^3^Yueyang Hospital Afiliated to Hunan University of Chinese Medicine, Yueyang 414000, Hunan, China

**Keywords:** aqueous humor, clinical therapy, intraocular pressure, pathological mechanism, Schlemm's canal

## Abstract

Schlemm's canal (SC) is a small circular canal in the deep part of the sclera at the junction of the sclera and cornea. As an integral component of the aqueous humor outflow, its structure and function are essential in regulating intraocular pressure (IOP). If SC develops lesions, the drainage of aqueous humor would be obstructed, leading to increased intraocular pressure and injury to the optic nerve. With the rapid development of minimally invasive glaucoma surgery, an increasing number of surgeons became familiar with SC, and the area generated substantial academic attention. The pathological mechanism and the therapy for SC that had been studied in recent years are summarized in this article, hoping to provide ideas for the treatment of glaucoma in the future.

## 1. Introduction

The production and circulation of aqueous humor are in dynamic equilibrium in a healthy human body. If the equilibrium was broken, abnormal intraocular pressure (IOP) would result. As a significant conduit in the aqueous outflow pathway, the SC was a circular canal composed of endothelial cells, a basement membrane, and a thin connective tissue layer. Lesions of this canal are an important trigger for the elevated IOP in glaucoma patients, thereby becoming a focal point in glaucoma treatment. This article reviews the mechanism and therapeutic advances of SC lesions.

## 2. Pathology Mechanism of SC

### 2.1. Pathological Changes of Endothelial Cells

The SC endothelium provides the only continuous cell layer between the anterior chamber and episcleral venous blood [1]. The aqueous humor penetrates endothelial cells through large vacuoles resembling sacs, on which small pores connect the inside and outside of the lumen [2] and the intercellular pores between the inside cells of adjacent SC walls [3].

However, the characteristics of the junction between SC and the juxtacanalicular tissue (JCT) adjacent to the trabecular meshwork (TM) enable a funnel effect in the biomechanical structure when the aqueous humor flows out. The funnel effect resulted from the funnel-shaped flow outlet generated by small pores. Thus, the endothelial cells were sufficiently porous to promote aqueous humor outflow and sufficiently restrictive to prevent blood and serum proteins from entering the eye through the blood-aqueous barrier [1]. Consequently, the increase in the density of small pores was most likely one of the causes of elevated IOP.

Normally, large vesicles are dynamic and increase in number and size with IOP [4]. At elevated IOP, the increased mechanical stress may adjust to the reorganization of the intermediate filament and the hardness of the cells by direct conduction of the intermediate filament or chemical conduction of changes in the activity of relevant enzymes. Stable IOP is maintained due to the increased expansion of large vacuoles created by endothelial cells toward the exterior of the subconjunctival space and the thinning of endothelial cell thickness, which tends to increase the number of tiny pores to drain the aqueous humor [5].

### 2.2. Pathological Changes of Extracellular Matrix (ECM)

Fibrosis of ECM enhanced resistance and the funneling effect of aqueous humor outflow. Recent studies have suggested that glaucomatous SC endothelial cells were stiffer and associated with reduced porosity and increased (ECM) material compared to SC endothelial cells from healthy individuals [6]. Specifically, the study found that SC endothelial cells in glaucoma deviated from typical characteristics and exhibited more fibrotic phenotypes. For example, significantly elevated levels of fibrosis markers *α*-SMA, type I collagen-*α* 1, fibronectin TGF-*β*2, endothelial cell proliferation and migration rate, and a decrease in mitochondrial activity were observed. Reina Torres et al. [7] also found that TGF-*β* activity expressed in endothelial cell fibrosis was also positively correlated with the deposition of ECM. From this perspective, influencing cellular protein expression to promote aqueous humor outflow was a potential therapeutic target for glaucoma.

### 2.3. SC Collapse

The pipe diameter of the SC also affects the resistance of the outflow of aqueous humor. Due to the influence of factors such as autonomic nervous system activity [8, 9], activation of the parasympathetic nervous system [10], senescence, and dysplasia [11], the diameter of the SC shrinks and collapses, resulting in the obstruction of aqueous humor outflow. Elevated IOP further compresses SC, producing irreversible deformation and lumen collapse with a smaller diameter [12]. Consequently, the decrease in SC diameter and the increase in IOP were mutually causative, generating a vicious cycle.

### 2.4. Obstructed Collector Channels

The pathologic lesion at the entrance and the cell layer on the inner wall of the SC impacted the outflow of aqueous humor through the collector channels. The morphology of the collector channel openings was elliptical in shape, commonly found in the mid-anterior planar region of the outer wall of the SC, and the complex orifices that were mostly septal columns and bridges connect the inner and outer walls of the SC and extend into a flap-like structure. The flap-like extension structure both serves as a tether to maintain the shape of the SC and a venous valve, preventing the backflow of aqueous humor entering the collector channels flowing back into the SC when the pressure in the SC was balanced with the extra scleral veins [13]. There was also a collagen structure at the edge of the collecting channels called a diaphragm column, moving with pressure changes, preventing the inner wall of the SC from protruding into the lumen of collector channels and maintaining SC patency [14].

## 3. Research Progress in Clinical Therapeutic Strategies

### 3.1. Progress in Medical Therapy

Elevated transforming growth factor beta2 (TGF-*β*2) in aqueous humor was associated with glaucomatous outflow dysfunction. The potential mediator of dysfunction was a yes-associated protein (YAP) and transcriptional coactivator with postsynaptic density protein 95/discs large/zonula occludens-1 (PDZ)–binding motif (TAZ). Li et al. [15] found that inhibition of YAP/TAZ attenuated the TGF-*β*2-induced dysfunction of human SC cells and had the potential to facilitate aqueous humor outflow.

Latanoprostene bunod (LBN) relaxed the tissue between TM and SC by binding with NO to assist in the efflux of aqueous humor [16, 17]. These medications shared similar principles, but the damage to the native structures of the eye was reduced over time, with fewer side effects and increased patient and physician acceptability. First-line medications that promote aqueous humor outflow through the uveoscleral pathway include FP-receptor prostaglandin (PG) agonists (e.g., latanoprost, travoprost, and tafluprost) and a novel non-PG EP2 receptor agonist (e.g., omidenepag isopropyl and eybelis) [18].

TM/SC efflux-enhancement drugs Rho-kinase inhibitors or rho-associated protein kinase inhibitors also effectively lowering IOP. RHO inhibitors possess neuroprotection properties, antifibrotic activity, endothelial cell proliferation effects, and lowing IOP [19]. These drugs had a synergistic effect when combined with others [20]. The mechanism could potentially be that ROCK inhibitors inhibit TGF-*β*2-induced endothelial-mesenchymal transition in SC cells and are related to p38 MAPK and BMP4 signaling pathways [21]. However, Rho-associated protein kinase inhibitors had a high incidence of congestion and low intraocular bioavailability when applied topically [22]. The ways in which to improve the safety and intraocular bioavailability of these medicines warrant further research.

Moreover, several studies concluded that topical epinephrine increased the diameter and area of SC, directly enlarging the channel for aqueous humor outflow to decrease IOP [23]. Nitric oxide-donating compounds directly targeting the TM/SC/conventional outflow pathway to reduce outflow resistance secured US Food and Drug Administration approval [24].

From this perspective, most glaucoma drugs that act on SC target the SC itself, endothelial cells, and JCT. All of them lowered the resistance of aqueous humor outflow, but the safety and bioavailability of their therapeutic application warrant further investigation.

### 3.2. Progress in Surgical Therapy

Due to its efficient IOP reduction, trabeculectomy has been commonly implemented in clinical settings. However, the medical procedure has more postoperative problems and is not always safe. In recent years, minimally invasive glaucoma surgery (MIGS) has become increasingly popular due to safety and consuming less time, with many MIGS procedures concentrating on SC.

#### 3.2.1. Trabecular Surgery

The surgical principle of trabeculectomy was to incise the inner wall of SC and TM, establishing a direct channel between the anterior chamber and SC and facilitating the flow of aqueous humor into SC and collector channels. The advantage of the surgery was that there was no filtration bleb. Therefore, there was no need to be concerned with scarring of the filtration bleb, causing an increase in IOP. However, it still had to contend with a major surgical incision and the potential for error in the position of the incised trabecular, which predisposed the formation of a false channel devoid of drainage abilities [25]. 360° trabeculectomy with an illuminated catheter filled with viscoelastic material to dilate SC [26], 360° and 180° SC incisions in suture trabeculotomy *ab interno* for open-angle glaucoma [27], gonioscopy–assisted transluminal trabeculotomy [28],and others were gradually spreading.

#### 3.2.2. Implantation Surgery

Canaloplasty *ab interno* formed a small goniotomy by viscous dilation of SC utilizing a medial approach with a transparent corneal incision under a gonioscope [29].

Viscocanalostomy and phaco-viscocanalostomy injected Viscoat™ (sodium hyaluronate and sodium chondroitin sulfate) into the surgically created ostia of SC, aiming at dilating both the ostia and the canal [30]. SC tubuplasty consists of surrounding the scleral venous sinus with an external tube and coupling it with a suture to expand the cross-sectional area of the SC [31]. Laser anterior chamber perforation surgery was also utilized to improve surgical success and prognosis.

There are also stent support surgeries such as iStent implant for internal surgery [32], iStent inject (Glaukos Corp.), and Hydrus Microstent [33, 34], which were effective options for the treatment of mild-to-moderate glaucoma due to their lower levels of damage to the ocular structures, ease of implantation, shorter surgical time and high safety [35].

### 3.3. Progress in Chinese Medicine

*Erigeron breviscapus* (Vant.)*Hand-Mazz* effectively promoted blood circulation, relaxed tendons, relieved pain, and eliminated stagnation. Its blood-stimulating function enhances the circulation of the aqueous humor. *Erigeron breviscapus* contains phenolic substances and their metabolites with antioxidant properties that efficiently protect optic nerve cells and SC endothelial cells [36].

*Salvia miltiorrhiza* Bunge has the effect of activating blood circulation, dispersing stasis, and relieving pain. The state of SC endothelial cells is an important factor in the smooth outflow of aqueous humor. The active components of *Salvia miltiorrhiza* Bunge, such as *Salvia miltiorrhiza* polysaccharides, inhibit inflammatory reactions and protect SC endothelial cells from pathological changes [37, 38].

*Lycium barbarum* nourishes the liver and kidneys. It had a positive therapeutic effect on glaucoma patients with liver and kidney shortage syndrome [39]. The *Lycium barbarum* polysaccharides could reduce intracellular reactive oxygen species (ROS) levels, inhibit the synthesis of nitric oxide synthase (NOS), and reduce the release of excessive NO [40], thereby alleviating the damage to SC endothelial cells.

## 4. Conclusion

Glaucoma was the first irreversible blinding eye condition in the world, and its mechanism of incidence and progression was extremely complex, with IOP being one of the key variables. As an important structure for the outflow of aqueous humor, the SC may serve as an entry point for regulating IOP. Medication and surgical procedures for SC had fewer side effects and a better prognosis than conventional treatment regimens. It was predicted that additional research would be conducted on SC once the pathogenic causes and appropriate treatments were well understood.

## Data Availability

No data were used for the research described in the article.
